# A Comparison of Mangrove Canopy Height Using Multiple Independent Measurements from Land, Air, and Space

**DOI:** 10.3390/rs8040327

**Published:** 2016-04-14

**Authors:** David Lagomasino, Temilola Fatoyinbo, SeungKuk Lee, Emanuelle Feliciano, Carl Trettin, Marc Simard

**Affiliations:** 1 Universities Space Research Association/GESTAR, 7178 Columbia Gateway Dr., Columbia, MD 21046, USA; 2 NASA Goddard Space Flight Center, Greenbelt, MD 20771, USA; 3 US Department of Agriculture, Forest Service, Cordesville, SC 29434, USA; 4 Jet Propulsion Laboratory, Pasadena, CA 91109, USA

**Keywords:** canopy height, DSM, biomass, Africa, H100, blue carbon, TDX, VHR, MRV

## Abstract

Canopy height is one of the strongest predictors of biomass and carbon in forested ecosystems. Additionally, mangrove ecosystems represent one of the most concentrated carbon reservoirs that are rapidly degrading as a result of deforestation, development, and hydrologic manipulation. Therefore, the accuracy of Canopy Height Models (CHM) over mangrove forest can provide crucial information for monitoring and verification protocols. We compared four CHMs derived from independent remotely sensed imagery and identified potential errors and bias between measurement types. CHMs were derived from three spaceborne datasets; Very-High Resolution (VHR) stereophotogrammetry, TerraSAR-X add-on for Digital Elevation Measurement, and Shuttle Radar Topography Mission (TanDEM-X), and lidar data which was acquired from an airborne platform. Each dataset exhibited different error characteristics that were related to spatial resolution, sensitivities of the sensors, and reference frames. Canopies over 10 m were accurately predicted by all CHMs while the distributions of canopy height were best predicted by the VHR CHM. Depending on the guidelines and strategies needed for monitoring and verification activities, coarse resolution CHMs could be used to track canopy height at regional and global scales with finer resolution imagery used to validate and monitor critical areas undergoing rapid changes.

## 1. Introduction

Mangroves may only represent 3% of the global forest cover, but it has been estimated that at the current rates of degradation, these forests can release up to 10% of the total carbon emissions from deforestation worldwide [1,2]. Beyond being one of the most carbon dense ecosystem brought upon by high carbon sequestration rates [1,3], mangrove forests are economically and biologically important both at local and global scales [4]. The large carbon stocks along with the many economical ecosystem services, high rates of degradation, and threats from rising seas, make mangrove environments important regions of interest for climate mitigation and adaptation plans [5]. Remote sensing can provide multiple independent techniques to monitor and verify forest parameters such as canopy height.

The benefits of measuring canopy height and biomass on a regular basis can help increase the transparency and accountability of local governments when considering programs similar to Reduction Emissions from Deforestation and Forest Degradation (REDD) and REDD+ [6–8]. These programs plan to incentivize the reduction of emissions and the development of forest retention [9], but capacity building is needed to accurately measure, monitor, and verify these carbon pools, particularly in the carbon-rich mangrove forests. In addition, intensive field surveys currently used to document forest carbon stocks, deforestation, and forest degradation from both natural human circumstances can be costly, time consuming, and dangerous. Incorporating remote sensing into Monitoring, Reporting, and Verification (MRV) frameworks will improve our understanding of the changes to forested ecosystems, increase the simplicity of aggregating local information to the global scales, enhance our precision and accuracy of models through multiple iterations, and verify results through multiple independent measurements.

Recent blue carbon studies have reported high rates of carbon burial and sequestration occurring among mangrove forest ecosystems [1,10]. The large mangrove carbon pool is a combination of the aboveground carbon production that also contributes to the stability of the much higher, belowground carbon sequestration [5]. Carbon and biomass estimates for mangrove forest vegetation are regularly derived from allometric equations that use parameters such as wood density, diameter-at-breast height (DBH), and tree height. The former attributes are difficult to estimate remotely, but mangrove canopy height can be derived from multiple computational techniques and from multiple aerial- and space-borne sensors [11–14]. Canopy height, in turn, can then be used to estimate ecosystem-scale above ground biomass using specific allometric equations [11,15,16]. However, current canopy height-based biomass estimates are still poorly constrained in these coastal forested ecosystems.

Mangrove forests provide a challenging ecosystem to accurately measure above ground carbon stocks because of the harsh physical conditions and tidal inundation that can inhibit access to surveyors. In order to efficiently and safely estimate carbon in the harsh pantropical coastal forests, various remote sensing techniques have been implemented. Three-dimensional maps of forest canopy heights have been generated using synthetic aperture radar (SAR), lidar, and high-resolution optical imagery. Airborne datasets acquired from lidar systems, like G-LiHT [17], can provide meter-to submeter spatial resolutions with height accuracies within 1.5 m for various forest types [18,19]. Though airborne lidar and similar systems are regarded as the “gold standard” for estimating canopy height, these flight campaigns can be expensive and cover limited regions. Data collected from space help to increase the area of interest to regional and global scales.

Spaceborne datasets from the Shuttle Radar Topography Mission and IceSAT/GLAS [11,12,16] have proven successful at estimating canopy height over mangrove forests at a relatively coarse spatial resolution of 90 m × 90 m resolution. Recently, global SRTM data have been released at a higher resolution of 30 m × 30 m. Other techniques like Polarimetric Synthetic Aperture Radar Interferometry (Pol-InSAR) have been applied to data collected from the TanDEM-X InSAR (TDX) mission to estimate canopy height [13,20]. High-resolution stereo-photogrammetry of IKONOS and WorldView1 and −2 imagery have also produced canopy height estimates at sub-meter spatial resolutions for boreal and temperate regions [21,22], and more recently, in mangrove forests [14].

There have been a number of remote sensing techniques employed to measure forest canopy heights from airborne and spaceborne platforms using lidar [12,18], stereophotogrammetry [21,22], and radar interferometry [11,13,23]. However, there has not been a comparison between each of these techniques at one site, particularly with respect to newer, modified, and higher resolution techniques such as Pol-InSAR and very-high resolution (VHR) satellite stereophotogrammetry. This paper will: (1) assess the utility of remote sensing to measure mangrove canopy height; (2) examine spatiotemporal variability between sensors; and (3) estimate errors and compare model efficiencies for mangrove canopy height models between independent remote sensing datasets.

## 2. Methodology

### 2.1. Study Area and Field Inventory

**Figure 1:**
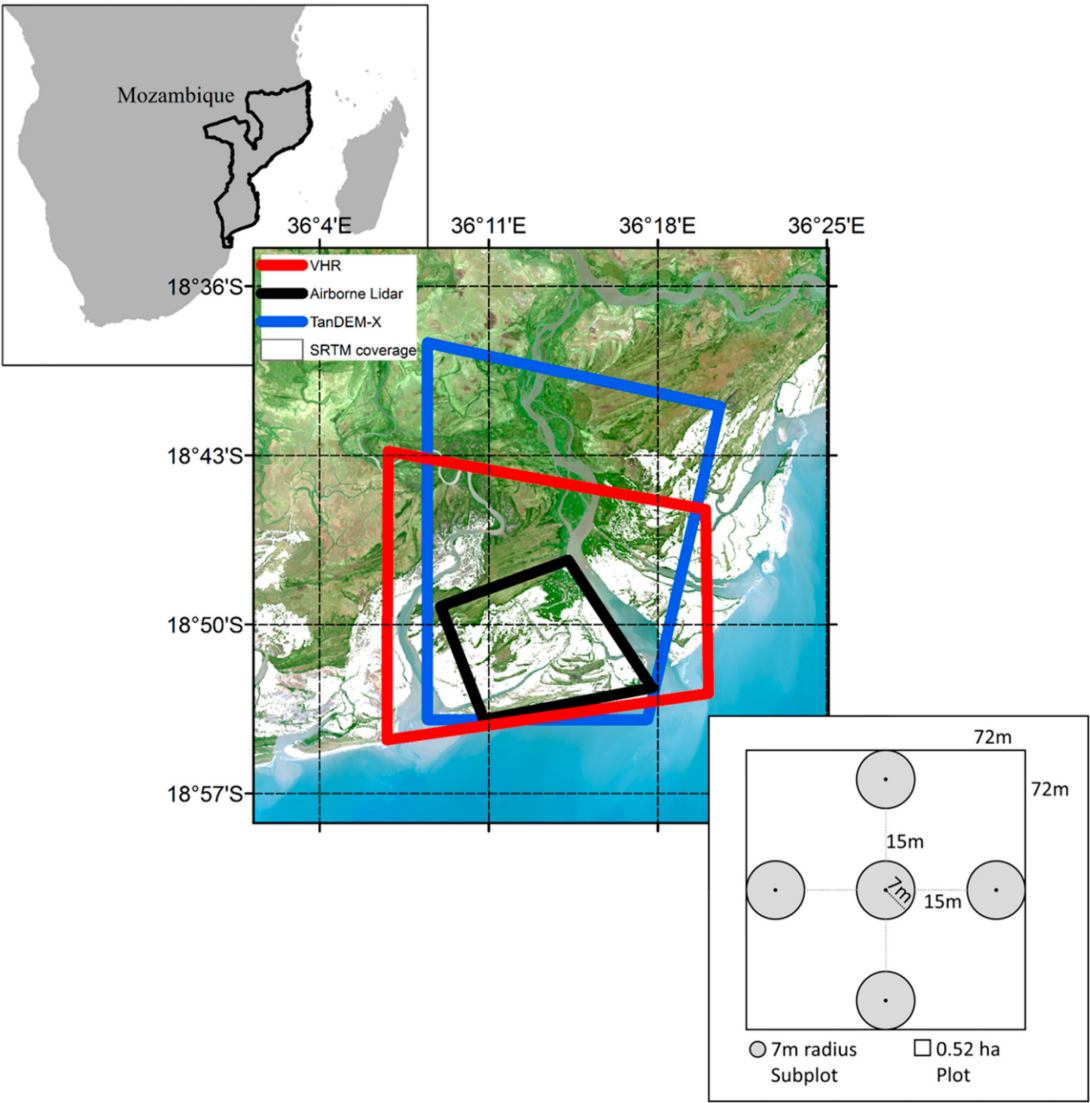
Location of the Zambezi Delta along the coast of Mozambique. Canopy Height Models (CHM) were generated over parts of the delta: airborne lidar (**black outline**); very-high resolution satellite imagery (**red outline**); TanDEM-X (**blue outline)**; and SRTM (**white area**). The field inventory plot design is shown in the lower right.

The Zambezi River sheds water from a 1,570,000 km^2^ area encompassing eight African countries and eventually discharges into the Indian Ocean via the Zambezi Delta (Figure 1). Distinct wet and dry seasons are present within this tropical region, with approximately 85% of the 1000 to 1400 mm annual rainfall occurring during the wet season from April to October [24,25]. Water that flows through the Zambezi Delta is balanced between the cumulative runoff from the large watershed and the semidiurnal tidal regime that can fluctuate up to 4.1 m twice a day [26]. Two dams constructed upstream in the mid-20th century, Kariba and Cahora Bassa, have modified seasonal river flows [27]. The damming has resulted in diminished seasonal stream flow signals [26,27], reduced sediment transport by 70%, and has changed the deltaic system from river-dominated to rain-dominated [28]. The sediments and water moving downstream are needed to support the coastal ecosystems of the delta, which include extensive grasslands, swamps, dunes, and mangroves [29].

An inventory of mangrove carbon stocks in the Zambezi Delta was conducted to provide a basis for inclusion of mangroves in the Mozambique national REDD+ strategy. The mangrove forest covers approximately 30,000 ha within the delta and is comprised of eight species: *Sonneratia alba, Avicennia marina, Rhizophora mucronata, Ceriops tagal, Bruguiera gymnorrhiza, Lumnitzera racemosa, Heritiera littoralis*, and *Xylocarpus granatum* [30].

In 2012 and 2013, a total of 52 (40 used in this study) inventory forest plots were measured to provide an unbiased estimate of the above- and belowground carbon stocks and determined that the biomass carbon densities ranged from 99.2 Mg· C· ha^−1^ to 341.3 Mg· C· ha^−1^ [30]. Only 40 plots were used in the present study because the other 12 were located outside the footprint of the remote sensing acquisitions. The inventory design incorporated recent recommendations for measuring carbon stocks in mangroves along with provisions to incorporate remote sensing, logistical constraints, and local information needs. The inventory design was based on a stratified random sampling method to improve precision of inventory results [30]. Field plots were stratified by mangrove canopy height maps derived from IceSat/GLAS and SRTM measurements from Fatoyinbo and Simard [12]. Canopy height, which is functionally related to biomass, was separated into five classes using natural breaks optimization: 2.0–7.0 m, 7.1–10.0 m, 10.1–13.0 m, 13.1–18.0 m, and 18.1–29.1 m [30]. Square 0.52 ha field plots were parsed into five smaller subplots each with a radius of 7 m (0.015 ha) to help account for the spatial variability within the larger plot (Figure 1). A central subplot was surround by additional subplots in each cardinal direction. Tree height, diameter at breast height (DBH), and species were recorded for trees in the overstory (DBH ≥ 5 cm) and understory (DBH < 5 cm). Tree height was measured using a hypsometer (Haglof Vertex III, Haglof Inc., Långsele, Sweden). Mean canopy height for the present study was estimated as the mean of all the overstory (DBH > 5 cm) trees in each subplot. The top of canopy height (H100), or the average of the 100 tallest trees per ha was calculated for each subplot. Since the measured area of the subplot was 0.015 ha, the average of the two tallest trees in each subplot represented H100. More detailed information about the sampling design and measurements can be found in Stringer *et al.* [30].

### 2.2. Canopy Height Models

Canopy height models (CHM) were collected and analyzed for the Zambezi Delta using multiple remote sensing platforms: airborne lidar, TerraSAR-X add-on for digital elevation measurements, high-resolution stereo-imagery, and SRTM.

#### 2.2.1. Airborne Laser/LiDAR Scanning

The vertical structure of forests has been successfully studied using airborne laser/lidar scanning (ALS) sensors [17,18]. Moreover, ALS is a proven technique to validate and calibrate vertical forest structure measurements acquired from spaceborne sensors (SRTM, TDX) in mangrove ecosystems [11,13,31]. To compare, enhance, and validate spaceborne-based assessments, ALS and multispectral data (NIR, Red, Green bands) were acquired 5–6 May 2014 by Land Resources International (Pietermaritzburg, South Africa). The airborne survey comprised an approximate area of 115 km^2^ in the Zambezi Delta (Figure 1) with a point density that ranged between 5 and 7 points· m^−2^. First ALS returns (canopy returns), which mark the location of tree canopies, were used to generate a 1 m × 1 m resolution mangrove Digital Surface Models (DSM). The DSM was generated using ENVI/IDL software by selecting and gridding the highest elevation value falling into each grid. Mangrove canopy heights were calculated relative to the Earth Gravitational Model 2008 (EGM2008) geoid, which provides a six-fold increase in resolution and an increased accuracy compared to EGM96 [32]. The mangrove DSM was georeferenced into a WGS84 datum and UTM Zone 36 South projection.

#### 2.2.2. TanDEM-X

The TanDEM-X (TerraSAR-X add-on for Digital Elevation Measurements) mission forms a pair of satellite instruments that enable single-pass interferometry to generate a consistent global digital elevation model (DEM) [33]. The TDX mission, for the first time, allows the acquisition of satellite polarimetric interferometric data at X-band without temporal decorrelation, which is the most critical factor for successful Pol-InSAR forest parameter estimation in conventional repeat-pass air-/space-borne SAR systems [34]. Single- and dual-pol spaceborne TDX data have been well-proven at estimating quantitative forest parameters over a tropical, temperate and boreal forest site by means of the Random Volume over Ground (RVoG) model, although X-band wavelength has been expected to have less sensitivity for vertical forest structure [35]. However, the single-pol TDX inversion can be applicable to the forest height inversion, if an external digital terrain model (DTM) is available for a forest test site. To overcome the limitation of single-pol mangrove forest application, Lee and Fatoyinbo [14] suggested estimating the ground (water) phase directly from the TDX interferogram with the assumption that the underlying topography over mangroves is negligible and flat due to the unique environment in which mangroves grow (*i.e.*, near the water mean level). This assumption reduces the amount of unknown variables in the RVoG model. The inversion approach has been successfully proven and has generated mangrove canopy height map at 12 m spatial resolution over Zambezi Delta, demonstrating the possibility to use TDX DEM acquisition to map mangrove height globally [13]. The TDX data used here were acquired on 14 October 2011 with a height of the ambiguity of −80.91 m. The swath of the TDX was about 32 km.

#### 2.2.3. Very High-Resolution Stereophotogrammetry

One pair of VHR stereoimages from WorldView1 (DigitalGlobe, Longmont, CO) was collected over the Zambezi Delta on 7 January 2013. The panchromatic image pair was acquired via an agreement with Digital Globe and the National Geospatial Intelligence Agency (NGA) [36]. The image pair was acquired in along-track setting to reduce confounding issues related to temporal decorrelation and sensor angles. In other words, the two images were collected in the same orbit using an optimized satellite viewing and sun angle geometry to improve accuracy and corrections. Parallax tie points were automatically derived using the NASA Ames Stereopipeline (ASP) 2.4 software, developed by the Ames Research Center in Mountain View, CA [37]. The user guide and program software are available at http://ti.arc.nasa.gov.

A digital surface model (DSM) of the Zambezi Delta was derived using the ASP program. An image correlation routine within the ASP matches similar pixels and calculates the distance between the focal plan and the Earth’s surface [38]. An affine adaptive window (subpixel mode = 2) was used to estimate the most accurate surface elevation relative to the WGS84 ellipsoid. The gridded resolution of the DSM was approximately 0.60 m × 0.60 m which was a function of the sensor viewing geometry of the original panchromatic images. Without using ground control points (GCP), a horizontal accuracy of 5.5 m or less was expected for the DSM as described by Hobi and Ginzler [39], regardless of land cover type.

Mangrove canopy heights estimated from VHR imagery in the Zambezi Delta were determined using similar methodologies as outlined in Lagomasino *et al.* [14]. Bare ground surfaces were identified on the VHR images and then overlaid on the VHR-CHM. Elevation values were then extracted from each of the identified ground surfaces and using the area of the ground surfaces a mean-weighted ground elevation of 0.13 m was calculated. Lastly, the mean-weight ground elevation was subtracted from the VHR-DSM, which resulted in the VHR-CHM.

#### 2.2.4. SRTM

Mangrove canopy height for the year 2000 was generated for Mozambique using SRTM data and validated with field measurements [16] and with GLAS footprint data by Fatoyinbo and Simard [12]. For this study, we generated an updated map of mangrove canopy height using the recently released 30 m resolution SRTM data and previously published calibration equations from Simard *et al.* [11]:

(1) $$H=2.1+0.84\;H_{\text{SRTM}}$$

where *H* is the weighted mean height of the canopy and *H_SRTM_* is the SRTM elevation. We then extracted and compared height estimates generated by SRTM with other remotely sensed CHMs over the Zambezi Delta. The RMS error of height estimates on Inhaca Island, a region in southern Mozambique, ranged from 2.4 to 3.6 m.

### 2.3. Comparative Analysis

Each CHM was resampled to 1 m × 1 m to match the spatial sampling of the airborne lidar dataset. The VHR was resampled using a bilinear interpolation, while SRTM and TDX datasets were downsampled using nearest neighbor. Similarly, all datasets were clipped to the extent of the lidar coverage and subsequently masked to the regions of mangrove tree cover identified by Giri *et al.* [40]. Each CHM covered an approximate mangrove area of 6118 ha. Both the mean and H100 canopy height were calculated for the field data, and lidar and VHR CHMs. The H100 reference height, or the average of the 100 tallest trees per ha, was determined by taking the maximum pixel value of a 10 m × 10 m moving window which corresponded to 0.01 ha [41]. Using this technique, we were able to determine the maximum height of the canopy that would be equivalent to the tallest tree within each subplot. A 7 m radial buffer was created around the center point of each field subplot and represents the approximate area surveyed for each subplot [30]. Canopy height data were averaged within each buffered subplots; the mean canopy height being derived from the average of the resampled CHM and H100 determined from the average of the resampled H100 map. Both mean and H100 canopy height estimates were derived for lidar and VHR CHMs while only TDX H100 top-of-canopy and SRTM InSAR phase center heights were considered in this study. Areas with bad pixels were removed from the analysis.

We used the airborne CHM as a reference to assess the spaceborne CHMs. All CHMs were validated with the *in situ* data. General statistics including the mean, median, and standard deviations were determined for each of the CHMs. A comparative analysis was performed through error and efficiency statistics, including root-mean-square-error (RMSE), the Nash–Sutcliffe efficiency, mean-absolute percent error (MAPE), and bias. The RMSE was used to determine the deviation between the measured (field and lidar) and modeled results, but the NMRSE was used to normalize the RMSE to the range of canopy height values. The Nash–Sutcliffe Efficiency (NSE) index is widely used in hydrologic studies to calculate model efficiency but can be sensitive to sample size, outliers, and bias [42]. NSE values close to a value of 1 represent better predictions of the actual values while values near 0 reflect predictions as accurate as the mean of the data. Negative NSE values suggest that the mean value is a better predictor than the model. The different model efficiencies provided the ability to compare the CHMs using different techniques to identify the limitations and strengths of each CHM. In addition, these modeling statistics contribute information regarding the strengths of models without the use of *p* values.

## 3. Results

First, we investigate overall canopy height statistics. Mean canopy height estimates generally show corresponding statistical properties while the H100 canopy height tend to exhibit more variability among mean, standard deviation, and median. The estimated mean canopy heights for the entire mangrove region were not significantly different between the field-measured, lidar and VHR CHM at 10.1, 10.76 and 10.95 m, respectively (Table 1). Statistics for mean canopy height were not derived for SRTM and TDX CHMs. Similarly, there was no significant difference between their standard deviations and only a small difference between the medians. The H100 CHMs reported much more variability with the average top-of-canopy height increasing from 10.72 m with SRTM to 15.25 m with lidar, and VHR and TDX CHMs falling in between the two (Table 1). The average and median H100 canopy heights were within less than 1 m for the VHR and lidar CHMs, though the lidar H100 heights were taller by 3 m. TDX and VHR CHMs also exhibited similar standard deviations, ~5.5 m, for both the mean and H100 models.

**Figure 2:**
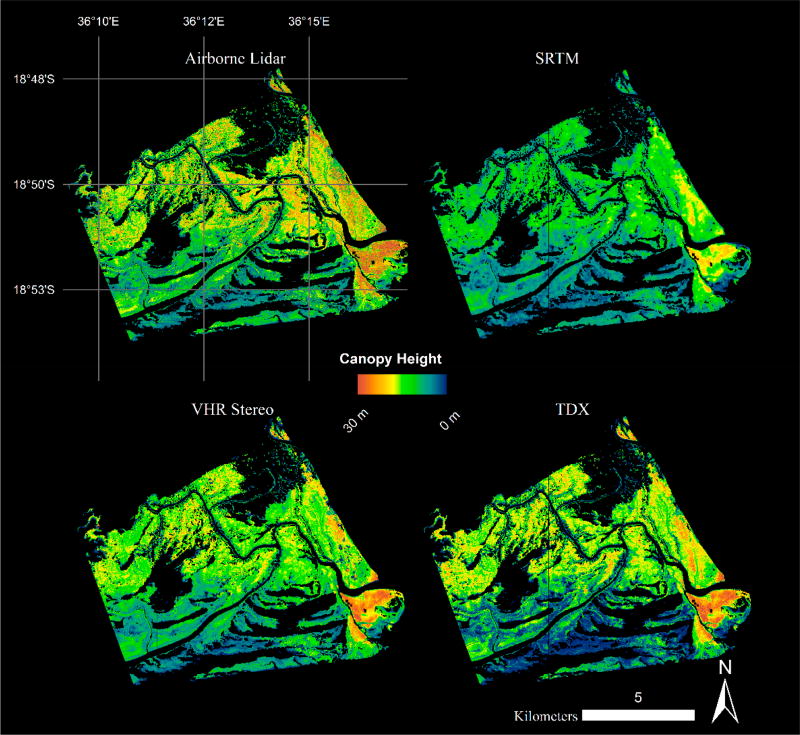
Four Canopy Height Models (CHMs) for a region of the Zambezi Delta (see black line on Figure 1 for region of interest): Airborne Lidar, Shuttle Radar Topography Mission, Very High Resolution (VHR) Stereo, and TanDEM-X (TDX).

**Figure 3:**
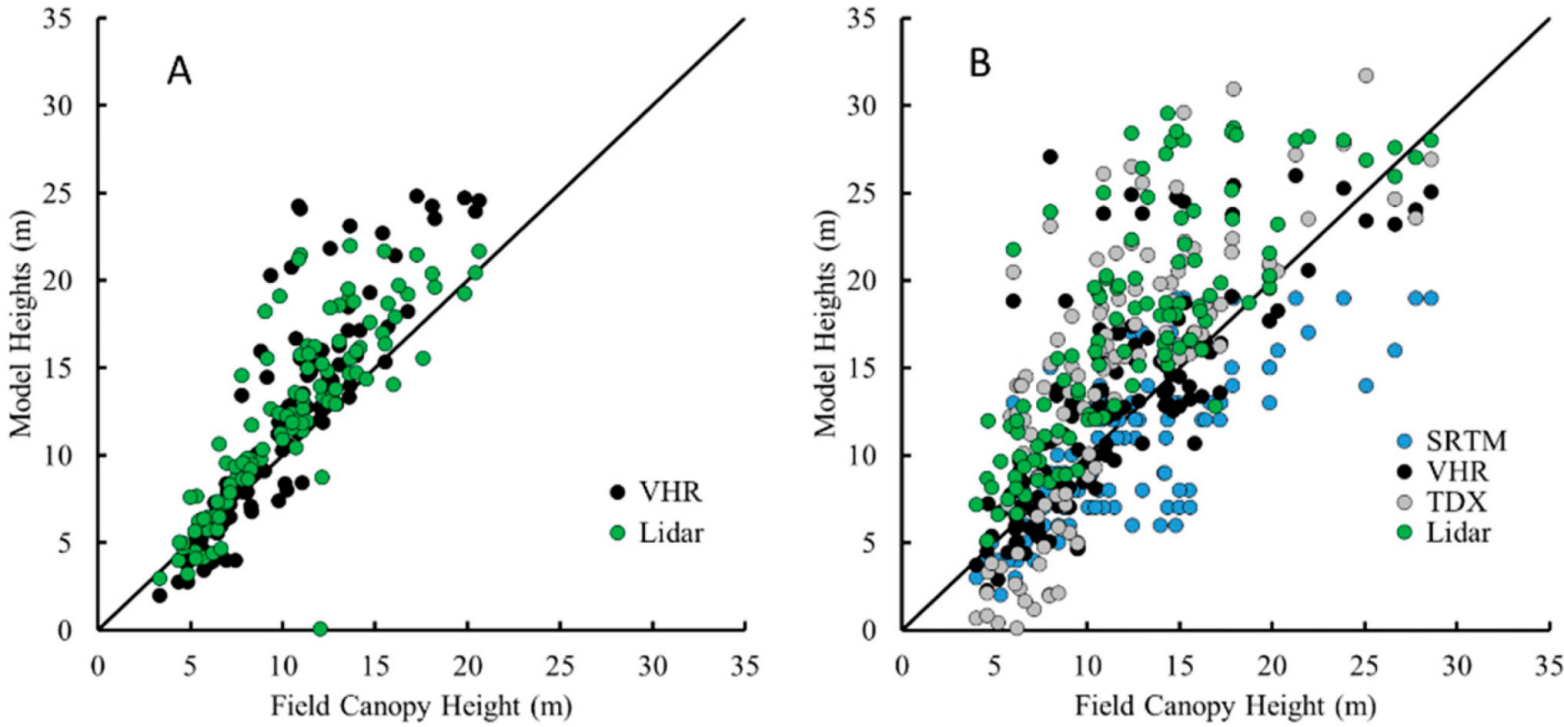
Relationship between model canopy height and field measured canopy height for each sensor at the subplot location: (**A**) comparions between the means; and (**B**) comparisons between H100.

Mangrove canopy heights determined at the subplot scale ranged between 1 and 30 m (Figures 2 and 3). Both the mean and H100 CHMs measured from remote sensing were positively correlated with mean and H100 field canopy heights (Figure 3). Mean and H100 CHMs overestimated actual canopy heights in areas where the *in situ* canopies were taller than 10 m, but overall still represented the study area field canopy height of 10.1 m (Table 1). More specifically, the mean VHR and lidar based approaches overestimated the canopy heights for canopies between 10 and 30 m and conversely, underestimated or provided better estimates of canopy heights in areas less than 10 m (Figure 3). Similar overestimation patterns were exhibited for the H100 CHMs for canopies greater than 10 m, but show different patterns for shorter canopies. In shorter canopy forests, the H100 lidar overestimated field canopies, while VHR and TDX CHMs underestimated the field top-of-canopy (Figure 3). The SRTM CHM consistently underestimated the H100 canopy for all heights. These estimates reflect the bias calculations where TDX had the strongest bias of 3.31–3.52 m followed by VHR with 1.33–1.88 m, then SRTM with 0.15–1.69 m (Table 2). The lidar CHM had a much more variable bias between the mean and H100 CHMs, 1.84 and 4.80 m, respectively.

The R^2^ values were similar amongst all the CHM models. Mean CHMs R^2^ values were 20% to 30% higher than H100 CHMs, ranging from 0.69 to 0.73 and 0.57 to 0.59, respectively (Table 2). SRTM CHM was a better predictor of field-derived mean canopy heights compared to the other remote sensing models despite the original 30 m × 30 m model being downscaled (nearest neighbor) to 1 m resolution. Error estimates, RMSE and MAPE, were highest for the TDX CHM, but remained consistent between mean and H100 models. The lowest errors were associated with the SRTM CHMs though there was a slight increase in the error from the mean to the H100 CHMs (Table 2). Lidar and VHR CHMs exhibited similar error statistics except for the lidar H100 model that was generally twice the error of the mean CHM.

**Figure 4:**
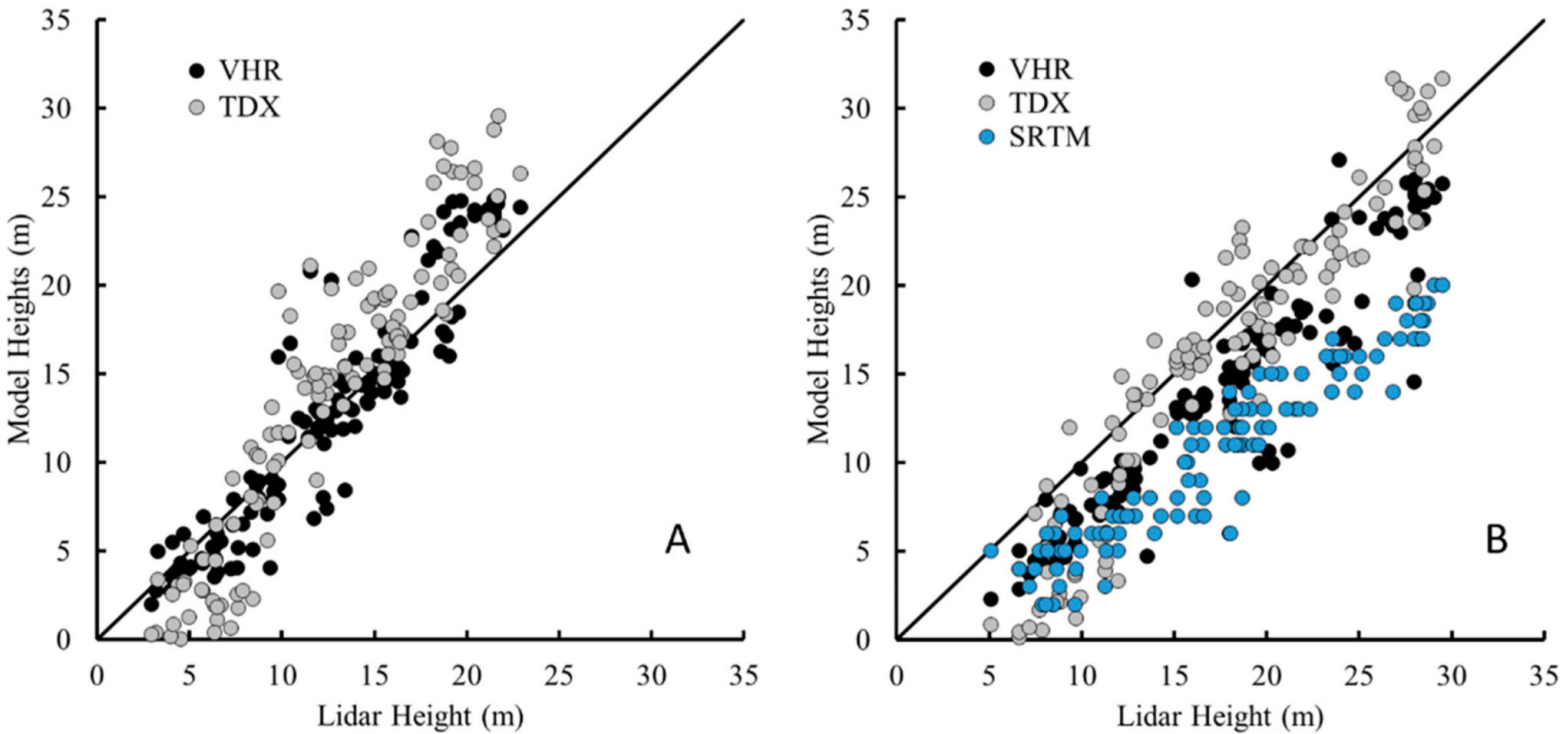
Relationship between airborne lidar measured canopy height and modeled canopy height for each sensor at the subplot location: comparions between the means (**A**); and comparisons between H100 (**B**).

Similar height relationships were exhibited for CHM comparisons between the field survey and lidar reference frames. There was as a notable increase in R^2^ values for all CHMs with the lidar reference compared to the field reference. R^2^ values increased to 0.82–0.90 and the RMSE dropped for all CHM except the SRTM CHM which resulted in an increase in RMSE (Table 3). As with the field reference, TDX canopy heights generally underestimated mean lidar canopy heights in mangrove stands less than 5–10 m tall. The VHR CHM indicated little to no bias while the TDX and SRTM CHMs exhibited a negative and positive bias respectively (Table 3). The NSE index was highest for VHR CHM_mean_ and TDX CHM_H100_ at 0.76 and 0.72, respectively. CHM_H100_ more precisely predicted top-of-canopy height for each subplot, though did show an increasing bias from TDX to VHR, and from VHR to SRTM. (Figure 4, Table 3).

**Figure 5:**
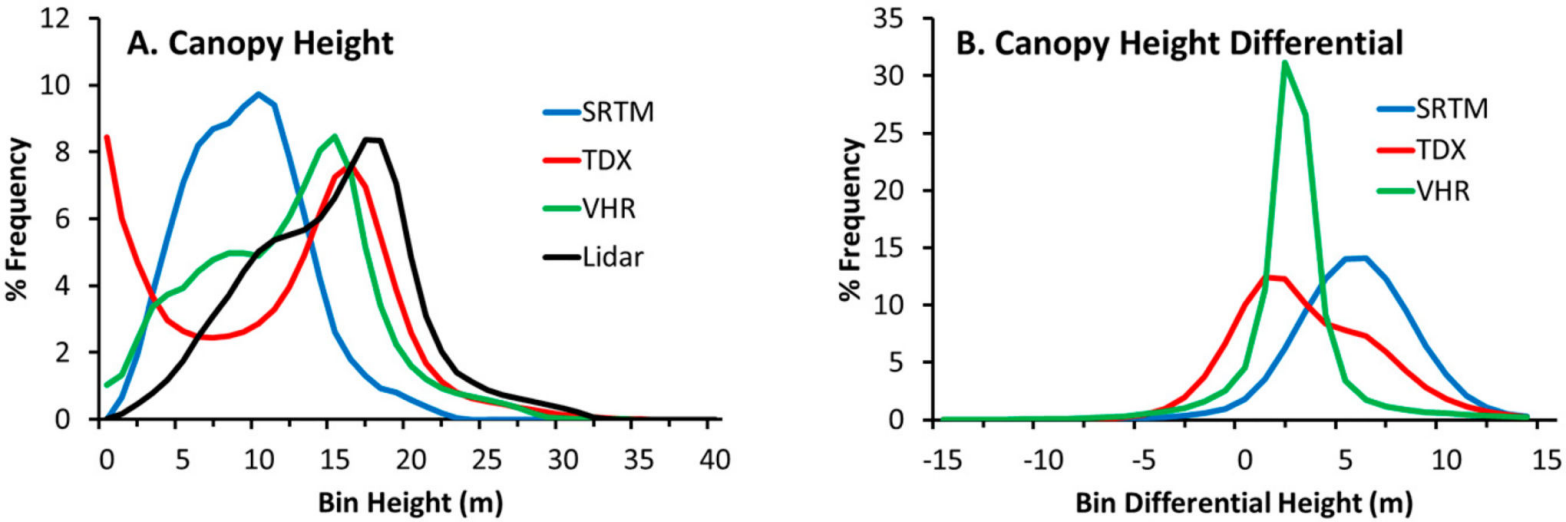
H100 canopy height (**A**) and canopy height differential (**B**) frequency distributions.

The H100 canopy height histogram distributions computed for all four CHMs (e.g., SRTM, TDX, VHR, and lidar) exhibit two distinct patterns: negatively skewed distributions with peak frequencies clustered around each other, and a positively skewed distribution with a lower magnitude frequency (Figure 5A). Lidar, TDX, and VHR CHMs all show similar maximum height frequencies between 15 and 18 m that accounted for nearly 8% of mangrove canopy in the study area. TDX canopy height values less than 5 m were removed from the distribution because of the high estimation errors within the shorter canopies that were associated with TDX data acquired at relatively small spatial baselines [14,35]. The VHR and lidar CHMs show near-identical distributions with an offset equal to the bias for the VHR CHM. More specifically, both distributions identify a distinct maximum peak and also depict a second canopy mode that was approximately 6–7 m shorter than the peak mode: 6–10 m for VHR CHM and 10–15 m for lidar CHM (Figure 5A).

Differential canopy heights, with respect to lidar, were also determined for satellite-derived CHMs. The TDX CHM has the lowest maximum frequency height differential at 1–2 m, followed by VHR CHM with 3 m, and finally SRTM with 6 m (Figure 5B). The mean difference between the lidar reference and satellite-derived CHMs for all mangrove pixels were 2.99, 3.58, and 6.06 m for VHR, TDX, and SRTM, respectively.

## 4. Discussion

### 4.1. Canopy Height Measurements

The results of the present study show a strong correlation between each of the datasets as seen in similar mangrove environments in the Caribbean [11,13] and Africa [14,16] as well as other deciduous and pine forest types [21,22,43]. The differences among CHMs can be related to the differences in ground reference frames (*i.e.*, field surveys and airborne lidar), spatial resolution, temporal resolution, time lag in acquisitions and senor sensitivities. Two ground reference frames, field surveys and airborne lidar, were used in this study to represent the ground-truthed canopy height. By comparing other independent remotely sensed CHMs (*i.e.*, SRTM, VHR, and TDX) with the field-measured and airborne lidar canopy heights we can discern the capabilities and height bias for each method.

The 0.015 ha subplots from the field survey were directly compared to overlapping areas on each CHMs. There were similar correlations between each of the different models, but with a relatively high initial RMSE that exceeded 3.4 m for all but one CHM. The error increased by 40%–50% when comparing the H100 parameter between the field measurements and the CHM_H100_ (Figure 3). In addition, the CHM mean was a much better predictor of the *in-situ* mean canopy height at the subplot level because the aggregation of trees in the overstory within each pixel is more consistent with the remote sensing measurement. More specifically, the lower mean canopy heights from SRTM best predicted the field values (NSE > 0.50). The penetration depth of the phase-centered canopy height elevation measured from the SRTM is partly a function of the tree canopy structure and density [11]. Lee and Fatoyinbo [13] reported a penetration depth up to 10 m in areas for InSAR X-band in the Zambezi Delta. C-band data from SRTM over French Guiana, exhibited a vegetation density-dependent canopy penetration range of 2.3 to 8.5 m compared to top-of-canopy measurements from RADARSAT but also include an overall elevation error of ±16 m that was complicated by ground topography [44]. As the radar signal interacts with the vegetation, changes in the over-and under-story canopy structure could drive the elevation of the phase center. The underestimation reported here for the SRTM CHM is most likely related to the C-band radar signal penetrating further through the mangrove canopy in the Zambezi Delta and may represent a relatively lower canopy height even after initial mangrove SRTM bias corrections [11].

Conversely, airborne lidar and VHR CHMs represent the top surface of the canopy. The commercial lidar data used in the present study were processed by the supplier based on first and last signal returns, and VHR imagery considers the optical properties reflected to the satellite. Clearly, changes in forest structure may have occurred since SRTM data acquisition (February 2000) and more recent datasets. In the years between SRTM and recent remote sensing acquisitions, there have only been six cyclones that have made landfall in Mozambique, with only Cyclone Funso indirectly affecting the study area [45]. Strong tropical storms will inevitably have an impact on mangrove forest as seen in other similar environments [46]; however, the major discrepancies between CHM would occur in localized areas. Although not in the scope of this study, large deviations between CHMs would suggest dramatic changes to the forest structure, indicative of clearing events from humans and cyclones.

There was no clear best predictor of field-measured canopy height with respect to R^2^ values, though SRTM CHMs did produce higher NSE values which did indicate better predictions of the actual mean field measurements collected in 2013 (Table 2). However, because of the larger spatial resolution of the SRTM imagery, many of the finer-scale changes in canopy height are overlooked and areas with the tallest trees are overlooked. In addition, a continental-scale SRTM correction algorithm was used that may not be suitable for more localized studies [12]. All other remotely sensed CHMs moderately estimated canopy height, including lidar with NSE values below 0.3. This phenomenon is most likely a result of the subplot scale at which the CHM estimates were integrated over. Variability in the spatial scales (*i.e.*, pixel size) of the CHMs can highlight differences in canopy height depending on the structure of the forest. Dense, homogenous canopies have been shown to remain relatively consistent across a range of pixel resolutions (e.g., 3 to 30 m). Forests with gaps tend to show more variability at fine spatial resolutions (<12 m) but become consistent at coarser resolutions (>12 m) [43]. This suggests the tree density and canopy closure of mangrove forests may impact variability in height estimates depending on which CHM technique is employed. It should also be noted that the geolocation accuracy of handheld GPS units decreases in remote areas under thick forest canopies, like those found in the Zambezi Delta. The lower geolocation accuracy of handheld units may result in poor coupling between field and image data.

The use of lidar in previous forest height studies, have proven the technique’s accuracy and reliability across forest types [18,43]. The small bias between the lidar CHM and field-measure values can be attributed to the averaging of shorter trees measured in the field to obtain a mean canopy height. However, because of its reliability, we compared all other remotely sensed datasets with the reference lidar acquisition with the goal of increasing model accuracy. Similar to the field data reference results, the lidar reference frame yielded similar positive correlations but with much higher R^2^ values, which were on average, 10%–30% higher than their respective field comparison (Table 3). The increase in model accuracy and efficiency based on the lidar reference frame suggests greater geolocation accuracy associated with high-resolution remote sensing data than geolocation with handheld GPS units. Improving the geolocation of field plots through surveys and differential GPS systems will most certainly improve field to remotely sensed canopy heights, but come at a financial cost. However, though geolocation may increase RMSE, it would not cause the biased trends (Table 3).

**Figure 6:**
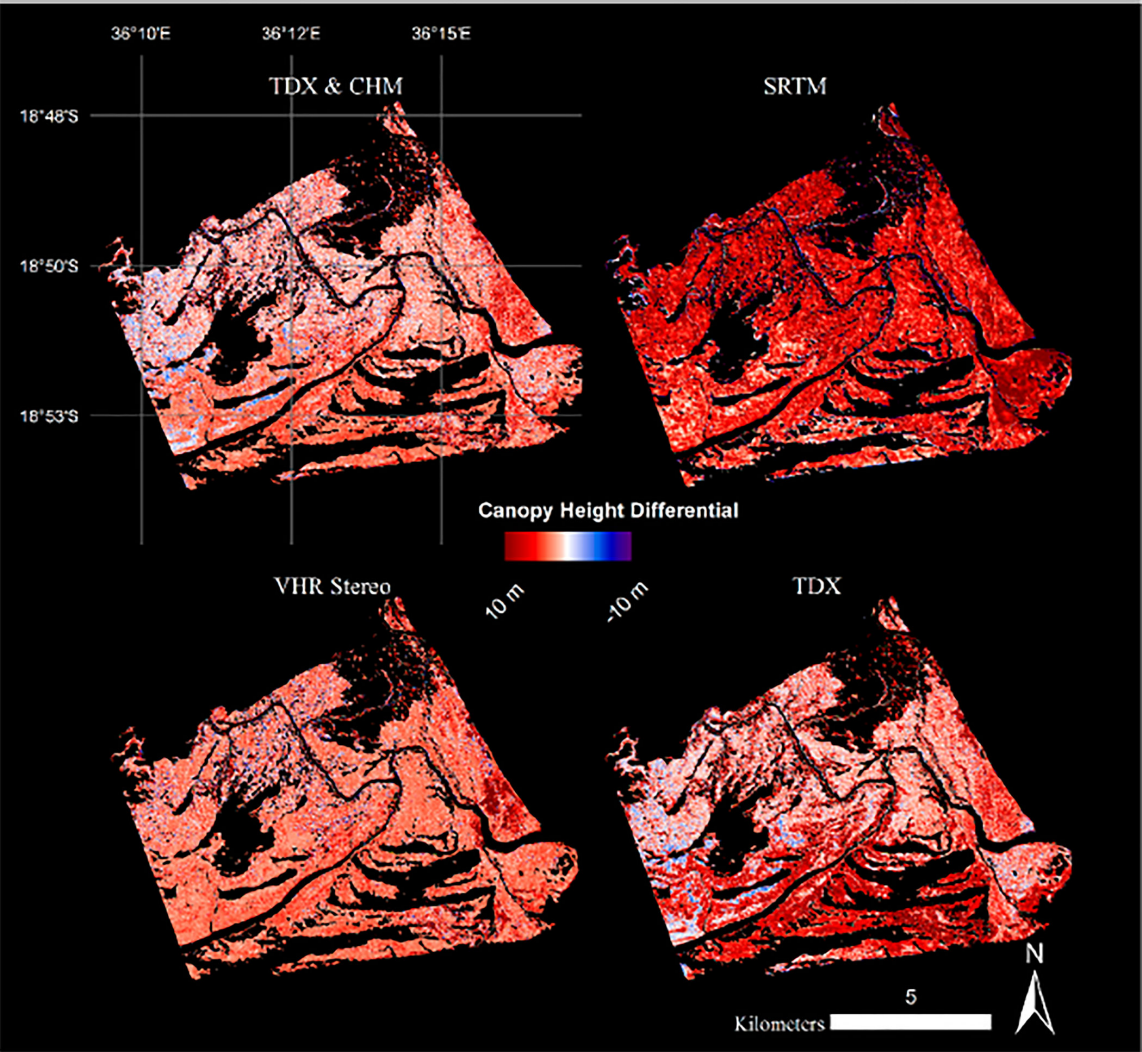
Canopy height differentials between reference airbone lidar and other Canopy Height Models (CHMs) for a region of the Zambezi Delta (see black line on Figure 1 for region of interest): Fused Very High Resolution (VHR) and TanDEM-X (TDX), Shuttle Radar Topography Mission (SRTM), VHR Stereo, and TDX.

The Pol-InSAR and stereophotogrammetry techniques used in this study have recently shown similar RMSE for measuring mangrove canopy height [13,14]. Lee and Fatoyinbo [13] calculated an RMSE of 1.3 m by comparing airborne lidar and TDX CHMs for a 1 ha over the Zambezi Delta, while Lagomasino *et al.* [14] determined an RMSE of 1.8 m by comparing field height to VHR CHMs for mangroves in Inhaca Island in southern Mozambique. Physical characteristics of the forest such as tree density, crown size and canopy structure may influence measurement accuracy and most likely depend on the instrument type. Understanding the sensitivities and biases of each of the CHM approaches will be particularly helpful to future canopy studies. According to previous studies, the TDX signal can be biased in shorter canopies as a result of a relatively small spatial baseline acquisition [13,47]. For a successful mangrove height inversion from TDX data over a wide range of mangrove forest heights, dual or multiple TDX acquisitions with variable spatial baselines (especially, larger baseline for small mangroves) may be required [34,47]. This study confirms the lower sensitivity by TDX for estimating shorter canopies, but performs best in canopies taller than 13 m (Figure 6). Conversely, VHR stereo derived CHMs estimated lidar CHMs with slightly better modeling efficiencies, but outperformed by TDX CHMs within canopies shorter than 13 m by nearly 100%. By combing the two techniques into a cross-platform CHM, at the 1 m × 1 m spatial resolution for the study, RMSE decreased by 15% and 31% compared to the original VHR and TDX CHMs (Table 4). More importantly, the NSE index also increased to 0.58, which indicates that the fused CHM predictions are better matched with the lidar observations than the VHR and TDX CHM that have a NSE index of 0.43 and 0.12, respectively.

When making comparisons between CHMs the reference elevation plane and the elevation of the measured physical parameter within the mangrove canopy also need to be considered. Because of the presumptuously flat terrain, we remove the underlying topography though differences between the geoid, ground and water level elevations, which could influence height variability between CHMs. The remote sensing approaches used in this study have variable elevation reference frames, which are a function of the data preprocessing and sensor sensitivities. Mangrove canopy height estimates generated from airborne lidar and SRTM were referenced to geoid models, while the TDX and VHR heights were fixed to physical references such as water level and the ground surface, respectively, after initial corrections to the geiod [13,14,16]. Small changes in frame of reference elevation may account for some of the differences reported in this study and actual canopy heights measured and reported across the Zambezi Delta [13].

Several factors need to be recognized and considered when comparing mangrove canopy heights from remote sensing. Forest type properties can drive differences in the CHM accuracies. For example, CHMs derived from SRTM data for pine forests tend to exhibit lower errors than for hardwood forest with a RMSE of 3.11 and 4.94 m, respectively, because of the forest crown structure [43]. Similar forest comparison between pine and hardwoods were reported by Neigh *et al.* [22] using VHR stereo CHM. Sparsely-dense larch forests, VHR CHMs underestimated field canopy height, but after calibration reduced RMSE to 1.37 m [21]. New CHMs techniques, similar to those used in the present study, have reduced initial RMSEs in SRTM CHMs from 3.55 [12] to 1.5 and 1.8 m using radar- and stereo-derived CHMs, respectively [13,14].

Next, the definition of canopy height should be specified. In our study we compared the mean height of the over-story canopy and the average of the tallest trees for each subplot. These two measurements represent two significantly different heights of the canopy as shown in this study. SRTM CHMs better predicted mean canopy heights measured in the field (Table 3) most likely as a result the penetration depth of C-band into the canopy and the fact that the mean field canopy height was an aggregation all of the overstory trees. Airborne lidar and optical sensors did not perform as well in estimated mean canopy height because they are more sensitive to the top of the canopy, and therefore the tallest trees.

Spatiotemporal effects also need to be considered when measuring mangrove forest structure over time. At relatively short timescales (e.g., tidal cycles and satellite flyovers), the assumptions for the flat terrain along mangrove coastlines that we presumed were static can, in actuality, change because of the sedimentation and erosion rates, and tidal fluctuations. During spring tides, water levels can reach an amplitude of 4 m in the Zambezi Delta. The ground phase of the TDX CHM was estimated by the ground, or water surface from a “double-bounce” and Pol-InSAR inversion. In microtidal coastal environments, where tidal fluctuations are less than 2 m, overall height estimates may not significantly change the overall distribution of mangrove canopy heights for the area. However, in areas where tides exceed 2 m, a positive or negative height bias may manifest in the CHM depending on the tidal cycle. A relatively easy response to removing any tidally influenced bias would be to correct TDX data with *in-situ* water level measurements.

Temporal changes in forest structure are also an attribute that needs to be considered when comparing CHMs. The four remote sensing datasets used in this study span 14 years, from 2000 to 2014, where SRTM data were collected in 2000, TDX data in 2011, VHR data in 2013, and the lidar data in 2014. Because of the discrepancies that could arise from using multi-temporal datasets, we parameterized our model comparison to consider overall changes in the mean and H100 canopy surface. Since H100 represents the tallest trees of the canopy, it also represents less change in height because of the lower mortality rates for trees with a DBH greater than 10 cm [48,49]. Therefore, our results confirm with other studies that suggest that canopy height comparisons between the H100 top-of-canopy heights permit a more robust long-term comparison by recognizing the lower mortality rates of taller trees and steady-state conditions in mature forests [14,50]. Previous studies have reported a less than 12% per year mortality rate for mangroves with a diameter-at-breast height (DBH) greater than 10 cm [48], and less that 3% per year for similar sized mangroves in protected equatorial waters around Malaysia [49]. In addition, two airborne lidar campaigns conducted in 1997 and 2006 for tropical forests in Costa Rica reported near-identical canopy height distributions and an average change of −0.32 m over the 8.5 year study [50], which is well within the error reported in the present study. Therefore, one of the major benefits of cross-platform forest monitoring would be detect areas of the canopy where substantial change has occurred (*i.e.*, deforestation). The change in canopy structure would identify areas of canopy loss or canopy growth greater than 2 m. In mature mangrove forests, large changes in canopy height or canopy cover are directly related to natural events like tropical storms or lightning strikes, or human intervention like mariculture or restoration.

### 4.2. Applications for Monitoring, Reporting, Verification

This study has compared several satellite remote sensing capabilities with forest inventory standard field and airborne lidar data. The comparison highlighted the similarities, errors, and biases between lidar, radar, and optical sensor types. VHR and TDX satellite provide an opportunity to deliver repeat measurements in order to better estimate forest canopy height, but more importantly, measure changes in the canopy over time. Canopy height has been shown to be directly related to biomass concentrations for mangrove forests [11,15,16]. Although we can predict mangrove forest biomass through these global equations, the accuracy of these estimates still needs to be refined locally.

Incentivized forest sustainability programs like UNREDD+ have specific precision and accuracy requirements. In order to meet these requirements, multiple remote sensing platforms can be used in harmony to assist with monitoring the three-dimensional structure of forest stands at the regional and global scales. Similarly, the verification process related to forest inventories can be through the comparisons of multiple remotely sensed CHMs. By understanding the biases and the reference measurements of SRTM, TDX, and VHR remote sensing datasets we can then make better interpretations regarding the landscape and refine our estimates to fall within forest inventory protocols.

Readily available satellite imagery can provide regional and global mangrove height estimates at relatively lower costs. The results from this study indicate that CHMs generated from several remote sensing techniques can provide precise estimates of mean and H100 canopy surfaces with a corrected RMSE of ~2 m. Because of the model efficiency using VHR and TDX, resources for lidar acquisitions can therefore be used more strategically to gather information in critical areas where changes in measurements would be less than 2 m. Critical areas where TDX or VHR data may not provide effective data would be areas that are degrading but not yet deforested or in areas where there is active canopy growth. The benefits of TDX and VHR CHMs will be their ability to document change in relatively mature forests or in forests undergoing rapid degradation. Adding remote sensing methodologies to current forest inventory standards will help achieve better regional estimates and complement field inventories by identifying areas of greatest canopy change.

### 4.3. Ecosystem Scale Modeling for Blue Carbon

The advancement of remote sensing technologies through increased sensor and spatial resolution capabilities provides an enormous amount of information to model ecosystems at local, regional, and global scales. In order to best capitalize on these techniques, and particular the fusion of the techniques, field inventories should also consider measurements that would benefit remote sensing estimates. This could include differential GPS and tree heights that are not routinely collected in forest inventories. Mangrove biomass estimates are primarily based on tree diameter, a parameter that is difficult to measure with satellite remote sensing. Developing robust allometric relationships to capitalize on remote sensing data and capabilities must be grounded in spatially and vertically explicit inventory surveys, not synoptic individual point measurements. Previous and planned mangrove forest inventories, particularly in Mozambique, Tanzania, and Gabon, will provide important advancements to incorporate cross-platform remote sensing as a decisive tool in the monitoring and verification of forest canopy height. In addition, the information on forest structure collected from similar mangrove studies can be incorporated into ecological function studies involving evapotranspiration [51], water quality [52], light-use-and water use-efficiency [53], carbon stock changes [54], and provide a new framework to refine regional carbon and water cycling.

## 5. Conclusions

We discussed the pros and cons of measuring mangrove canopy height at large spatial scales using various remotely sensed datasets. More importantly, we have assessed the accuracy of two relatively new high-resolution satellite derived CHMs (e.g., TDX and VHR) and three proven forest canopy methodologies: field surveys, SRTM, and airborne lidar. Our results show a strong correlation between each of the datasets. For certain regions, field data can be an expedited and economical method to measure canopy height at local scales. However, the remoteness and harsh conditions in mangrove forests prohibit the efficacy of such field campaigns. Therefore, remote sensing can play a crucial role in measuring baseline conditions and continuously monitoring mangrove forests at regional and global scales to help inform better management practices and provide verification for incentivized carbon programs similar to REDD+.

Several factors beyond costs and coverage should be considered when selecting a particular technique to estimate canopy heights in mangrove forests: spatial resolution, sensor sensitivities, and reference frames. Coarse 30 m × 30 m resolution SRTM imagery provides global coverage of mangrove canopy heights and generally predicts the mean overstory canopy height. The time lag between remote sensing acquisitions provides some discrepancy, because of canopy growth that may have occurred. SRTM data therefore poorly represents the top of the canopy, overlooks fine-scale forest canopy properties that are needed for more localized studies. Conversely, SRTM may be more stable overtime because changes at finer resolutions (e.g., gap dynamics) do not interfere so much with measurement and can accurately represent mean canopy height and canopy height distribution in mature, intact mangrove forests. In addition, significant changes in canopy height between SRTM canopy models and more recent models by signal deforestation and may augment land cover change research. TDX mangrove CHMs provide some of the highest spatial resolution radar altimetry estimates to date and has global coverage. Height estimates among the taller mangrove canopies are accurately represented using TDX but fail to detailed estimates for shorter canopies because of the baseline and height ambiguity issues. Though current efforts are being made to adjust the baseline in order to estimate the height of lower canopy vegetation. Lastly, VHR CHMs derived from stereophotogrammetry provided the lowest RMSE and highest NSE values on a pixel-by-pixel basis with the “gold standard” of forest canopy models, airborne lidar.

Depending of the scope of future studies and applications, aggregating canopy heights into discrete height classes could be used to reduce the errors across the height classes because of the inherent variability between different CHMs associated with spatial resolution and reference frames. Ultimately, reducing the error between classes will help in meeting carbon and restoration guidelines and protocols.

Future mangrove forest mapping applications will be augmented by the high-resolution CHMs described here to refine biomass estimates, but will inevitably be enhanced when combined with other mapping techniques like texture analyses, tree density, and spectral classification.

## Figures and Tables

**Table 1:** General statistics for mean and H100 field-measured tree heights and Canopy Heights Models (CHM) generated with different remote sensing platforms. Units for canopy height are in meters. Mean canopy was determined for field, lidar, and Very High Resolution (VHR) imagery. H100 canopy heights were determined for field, lidar, VHR, Shuttle Radar Topography Mission, and TDX (TanDEM-X). SRTM and TDX canopy height models were originally processed for the top-of-canopy and therefore, an average canopy height was not determined.

	Mean Canopy				H100 Canopy			
	
	Field	Lidar	VHR	Field	Lidar	VHR	SRTM	TDX
Mean	10.1	10.76	10.95	14.99	15.25	12.26	10.72	11.67
SD	3.4	5.4	5.44	5.87	5.39	5.59	2.16	7.15
Median	10.2	10.78	11.38	14.9	15.67	12.8	11	13.3

**Table 2:** Modeling statistics for mean and H100 model comparison between field data and remote sensing approaches at each subplot. Model efficiencies were compared between field and remote sensing values of the mean and H100, respectively. For Shuttle Radar Topography Mission (SRTM) and TanDEM-X (TDX) Canopy Height Models (CHMs), H100 estimates were used in both the field-derived mean and H100 values.

Field Reference								

	VHR		SRTM		TDX		Lidar	

	Mean	H100	Mean	H100	Mean	H100	Mean	H100
R^2^	0.73	0.57	0.69	0.57	0.70	0.57	0.71	0.59
RMSE	3.97	4.30	2.52	3.87	5.78	6.11	3.41	6.40
MAPE	0.24	0.28	0.20	0.24	0.49	0.48	0.23	0.46
NSE	−0.19	0.31	0.55	0.46	−1.45	−0.40	0.25	−0.39
Bias	−1.83	−1.33	0.15	1.69	−3.31	−3.52	−1.84	−4.80

R^2^= coefficient of determination; RMSE = Root Mean Square Error; MAPE = Mean Absolute Percent Error; NSE = Nash–Sutcliffe Efficiency Index.

**Table 3:** Modeling efficiency statistics for mean and H100 model comparison between lidar and other remote sensing approaches at each subplot. Model efficiencies were compared between field and remote sensing values of the mean and H100, respectively. For Shuttle Radar Topography Mission (SRTM) and TanDEM-X (TDX) Canopy Height Models (CHMs), H100 estimates were used in both the field-derived mean and H100 values.

Lidar Reference						

	VHR		SRTM		TDX	
	
	Mean	H100	Mean	H100	Mean	H100
R^2^	0.87	0.88	0.82	0.90	0.87	0.88
RMSE	2.57	4.20	3.19	7.32	3.93	3.48
MAPE	0.17	0.23	0.24	0.41	0.30	0.21
NSE	0.76	0.60	0.65	−0.16	0.44	0.72
Bias	−0.12	3.52	2.24	6.88	−1.36	1.63

R^2^= coefficient of determination; RMSE = Root Mean Square Error; MAPE = Mean Absolute Percent Error; NSE = Nash–Sutcliffe Efficiency Index.

**Table 4:** Modeling efficiency statistics for H100 model comparison between all pixels of lidar and other remote sensing approaches.

	Fused VHR-TDX	VHR	TDX	SRTM
R^2^	0.47	0.47	0.47	0.47
RMSE	3.49	4.08	5.06	6.78
MAPE	0.23	0.26	0.34	0.42
NSE	0.58	0.43	0.12	−0.58
Bias	2.2	2.99	3.58	6.06
